# Supplementary material for: Defining human mesenchymal and epithelial heterogeneity in response to oral inflammatory disease
Source: eLife. 2021 Jan 4;10:e62810. doi: 10.7554/eLife.62810 (PMC7781605; doi:10.7554/eLife.62810)
Supplement: Transparent reporting form [file elife-62810-transrepform.docx]

***eLife’s* transparent reporting form**

We encourage authors to provide detailed information *within their submission* to facilitate the interpretation and replication of experiments. Authors can upload supporting documentation to indicate the use of appropriate reporting guidelines for health-related research (see [EQUATOR Network](http://www.equator-network.org/%20)), life science research (see the [BioSharing Information Resource](https://biosharing.org/)), or the [ARRIVE guidelines](http://www.plosbiology.org/article/info:doi/10.1371/journal.pbio.1000412) for reporting work involving animal research. Where applicable, authors should refer to any relevant reporting standards documents in this form.

If you have any questions, please consult our Journal Policies and/or contact us: [editorial@elifesciences.org](mailto:editorial@elifesciences.org).

**Sample-size estimation**

- You should state whether an appropriate sample size was computed when the study was being designed
- You should state the statistical method of sample size computation and any required assumptions
- If no explicit power analysis was used, you should describe how you decided what sample (replicate) size (number) to use

Please outline where this information can be found within the submission (e.g., sections or figure legends), or explain why this information doesn’t apply to your submission:

We followed standard methods for single-cell studies of human tissues.

No statistical method was used to predetermine sample size.

**Replicates**

- You should report how often each experiment was performed
- You should include a definition of biological versus technical replication
- The data obtained should be provided and sufficient information should be provided to indicate the number of independent biological and/or technical replicates
- If you encountered any outliers, you should describe how these were handled
- Criteria for exclusion/inclusion of data should be clearly stated
- High-throughput sequence data should be uploaded before submission, with a private link for reviewers provided (these are available from both GEO and ArrayExpress)

Please outline where this information can be found within the submission (e.g., sections or figure legends), or explain why this information doesn’t apply to your submission:

- Four scRNA-seq datasets were generated for the human gingiva.
- Histological and immunofluorescence microscopy studies were performed on at least 3 human specimens per condition.
- Single-cell sequencing data quality control (data exclusion) is extensively explained in the Methods section of the manuscript. It follows the standards in the field.
- Single-cell RNA sequencing data from this study have been deposited in the Gene Expression Omnibus (GEO) under the accession number GSE152042.

**Statistical reporting**

- Statistical analysis methods should be described and justified
- Raw data should be presented in figures whenever informative to do so (typically when N per group is less than 10)
- For each experiment, you should identify the statistical tests used, exact values of N, definitions of center, methods of multiple test correction, and dispersion and precision measures (e.g., mean, median, SD, SEM, confidence intervals; and, for the major substantive results, a measure of effect size (e.g., Pearson's r, Cohen's d)
- Report exact p-values wherever possible alongside the summary statistics and 95% confidence intervals. These should be reported for all key questions and not only when the p-value is less than 0.05.

Please outline where this information can be found within the submission (e.g., sections or figure legends), or explain why this information doesn’t apply to your submission:

Statistical information is provided within the figures and their corresponding legends.

Methodology is detailed in the Methods section.

(For large datasets, or papers with a very large number of statistical tests, you may upload a single table file with tests, Ns, etc., with reference to sections in the manuscript.)

**Group allocation**

- Indicate how samples were allocated into experimental groups (in the case of clinical studies, please specify allocation to treatment method); if randomization was used, please also state if restricted randomization was applied
- Indicate if masking was used during group allocation, data collection and/or data analysis

Please outline where this information can be found within the submission (e.g., sections or figure legends), or explain why this information doesn’t apply to your submission:

Group allocation is provided in detail in the Methods section.

Briefly, age-matched healthy specimens were collected from patients with no history of periodontal disease; age-matched diseased specimens were collected from patients undergoing surgical periodontal treatment thus diagnosed with chronic periodontitis.

**Additional data files (“source data”)**

- We encourage you to upload relevant additional data files, such as numerical data that are represented as a graph in a figure, or as a summary table
- Where provided, these should be in the most useful format, and they can be uploaded as “Source data” files linked to a main figure or table
- Include model definition files including the full list of parameters used
- Include code used for data analysis (e.g., R, MatLab)
- Avoid stating that data files are “available upon request”

Please indicate the figures or tables for which source data files have been provided:

- Source data is provided for figures 1, 3 and 4.
- The following software has been used for data collection: Cell Ranger Single-Cell Software Suit (v.2.1.1, 10x Genomics), BD FACS Diva, Nanozoomer-XR Digital slide scanner (Hamamatsu), LAS X Leica, Graphpad Prism.
- The following software has been used for data analysis:

scRNA-seq analysis:

- R code sources;
- Seurat v3.2.1: <https://github.com/satijalab/seurat>
- Enrichr: https://maayanlab.cloud/Enrichr/
- NicheNet: <https://github.com/saeyslab/nichenetr>
- CytoTRACE: <https://cytotrace.stanford.edu>

Image analysis:

- ImageJ v1.52; Adobe Photoshop 21.1.2; Adobe illustrator v24.3.0.

Raw sequencing data is currently stored in NCBI/GEO datasets under accession GSE152042. These will be made publicly available upon acceptance of the manuscript.
